# Genetic heterogeneity in breast cancer: the road to personalized medicine?

**DOI:** 10.1186/1741-7015-11-151

**Published:** 2013-06-18

**Authors:** Richard D Baird, Carlos Caldas

**Affiliations:** 1 Department of Oncology, University of Cambridge, Addenbrooke’s Hospital, Hills Road, Box 97, Cambridge CB4 3EJ, UK; 2 Cancer Research UK Cambridge Research Institute, Li KaShing Centre, Robinson Way, Cambridge CB2 0RE, UK

**Keywords:** Breast cancer, Next-generation sequencing, Whole-genome sequencing, Molecular profiling, Personalized medicine, Heterogeneity

## Abstract

More women die from breast cancer across the world today than from any other type of malignancy. The clinical course of breast cancer varies tremendously between patients. While some of this variability is explained by traditional clinico-pathological factors (including patient age, tumor stage, histological grade and estrogen receptor status), molecular profiling studies have defined breast cancer subtypes with distinct clinical outcomes. This mini-review considers recent studies which have used genomics technologies in an attempt to identify new biomarkers of prognosis and treatment response. These studies highlight the genetic heterogeneity that exists within breast cancers in space and time.

## The genetic heterogeneity seen in breast cancer has important clinical implications

In 2008 it was estimated that the annual number of new breast cancer diagnoses globally was approximately 1.4 million, with just under half a million deaths. It has long been recognized that the clinical course of breast cancer varies tremendously between patients. Traditional clinico-pathological variables, including tumor stage, grade and estrogen receptor status, have been used for decades by clinicians to help prognosticate and guide treatment of their patients. In the last 30 years or so, a range of molecular biology technologies, including gene expression profiling, have been used to define molecular subgroups of breast cancer with distinct clinical outcomes [1-3]. These studies have identified recurrent somatic abnormalities, including gene mutations, copy number aberrations and translocations, the most important of which has been the *ERBB2* amplification present in 15 to 20% of breast cancers [4]. This mini-review considers recent studies that have used genomic technologies in an attempt to identify new biomarkers of prognosis and treatment response for patients with breast cancer.

## Recent next-generation sequencing studies

Whole-genome sequencing studies have reported tens of thousands of somatic mutations in different cancers [5-7]. The evidence suggests that only a small minority of these are essential for cancer development (“driver mutations”) with the majority having no significant biological impact (“passenger mutations”) [8]. In the clonal evolution model of malignant progression first proposed in 1976 by Nowell [9], different cancer clones within a tumor are in constant competition, with the “fittest” clones proliferating at the expense of “less fit” clones. Key driver mutations are thought to provide a selective advantage on a cell to facilitate its clonal expansion [9].

The degree of genetic heterogeneity within tumors from individual patients in both space and over time is increasingly well characterized [10]. In one early report using whole-genome sequencing, Shah *et al*. examined paired, metachronous tumors from a single patient with advanced invasive lobular carcinoma of the breast, and found 19 non-synonymous mutations present in metastatic tumors that were not evident in the primary tumor diagnosed nine years earlier [11].

Nik-Zainal *et al*. characterized the molecular profiles of 21 primary breast cancers in terms of copy number changes, genomic rearrangements and whole genome sequencing [12,13]. The authors used “most-recent common ancestor” bioinformatics algorithms to infer changes in mutation signatures over time. A key finding from these studies was that each tumor contained a dominant clone (>50% of cancer cells) which had a mutational profile very different from those of other sub-clones [12].

Shah *et al*. examined genome aberrations in a series of 104 primary “triple-negative” breast cancers using Affymetrix SNP6.0 arrays, RNA-seq and genome/exome sequencing. These tumors are so called “triple-negative” because they lack estrogen receptor, progesterone receptor and *ERBB2* amplification [14]. This study showed beyond doubt that this “catch-all” diagnosis of exclusion in fact refers to a group of cancers that exhibit great genetic heterogeneity. Interestingly, the abundance of somatic mutations in a given tumor did not correlate with the proportion of the genome with copy number alterations (CNAs).

Stephens *et al*. analyzed the genomes of 100 tumors for copy number alterations and mutations in coding exons of protein-coding genes [15]. The authors found correlations among the number of somatic mutations, the age at which cancer was diagnosed and tumor histological grade. New driver mutations were found in nine cancer genes including: *AKT2, ARIDIB, CASP8, CDKN1B, MAP3K1, MAP3K13, NCOR1, SMARCD1*and*TBX3*[15].

Banerji *et al*. focused on the use of whole exome sequencing to identify patterns of mutation and translocation from 103 breast cancers from a range of subtypes [16]. The authors confirmed the presence of *PIK3CA*, *TP53*, *AKT1*, *GATA3* and *MAP3K1* mutations, but also identified a recurrent *MAGI3*-*AKT3* fusion found most commonly in ER/PR-negative, HER2-negative breast cancers. Functional experiments showed that this fusion gene caused constitutive activation of AKT kinase which was amenable to therapy with a selective, small-molecular AKT inhibitor [16].

In the largest breast cancer series reported to date, the METABRIC study group performed an integrated analysis of copy number and gene expression in discovery and validation sets each containing approximately 1,000 primary breast tumors, with long-term clinical follow-up [17]. Inherited genetic variants (single nucleotide polymorphisms (SNPs) and copy number variants (CNVs)), and acquired somatic CNAs were associated with altered gene expression in approximately 40% of genes. Importantly, analysis of the combined DNA-RNA profiles revealed 10 different sub-groups with distinct clinical outcomes, which reproduced in the validation cohort. These included subgroups not previously identified by first-generation gene expression profiling studies, in particular with seven distinct subtypes of ER positive disease and a separation of triple negative cancers into at least two subtypes [1]. Indeed, there is increasing evidence that diagnosis of “triple negative” breast cancer does not describe a single biological entity with distinct natural history. Rather, it refers to a wide range of cancers with great genetic diversity, which can be further classified into multiple subtypes [18]. In one study, the functional heterogeneity observed within the stem-cell-like compartment of triple-negative breast cancers revealed a 31-gene signature which was associated with the development of metastatic disease [19].

In addition to studies using genomic techniques to identify prognostic biomarkers, a number of studies are emerging focused on the identification of biomarkers that predict response to therapies. For example, Ellis *et al*. performed whole exome and whole genome sequencing on 31 and 46 samples collected in two neoadjuvant aromatase inhibitor trials [20]. The most significant such finding was that mutant *GATA3* appeared to correlate with treatment-induced anti-proliferative effect [20].

The most recent large breast cancer sequencing study to be published is that of the Cancer Genome Atlas Network [21]. The investigators analyzed tumor and germline DNA samples from 825 primary breast cancers using orthogonal techniques, with different subsets of patients assayed on each of the following platforms: genomic DNA copy number arrays, DNA methylation, exome sequencing, mRNA arrays, microRNA sequencing and reverse-phase protein arrays. Analysis of the combined data from five platforms suggested there were four main breast cancer classes, with each of these subgroups characterized by significant molecular heterogeneity. Once again this study confirmed that there were relatively few “high-frequency” somatic mutations, with only three genes (*TP53*, *PIK3CA* and *GATA3*) occurring at >10% incidence across all breast cancers. Table 1 summarizes the most common mutations found in recent large sequencing studies of breast cancer. Intriguingly, comparison of basal-like breast tumors with high-grade serous ovarian cancers uncovered many molecular similarities. The authors concluded that “much of the clinically observable plasticity and heterogeneity occurs within, and not across, the major biological subtypes of breast cancer”. However, when the cancers were classified into the 10 subtypes identified in METABRIC there were clear patterns of cluster-specific mutational landscapes emerging, providing strong support for the new molecular taxonomy of breast cancer.

**Table 1:** Most frequently mutated breast cancer genes

		**Approximate mutation frequency (%)**				
**Gene mutation**	**Function**	**Overall**	**Luminal A**	**Luminal B**	**HER2-enriched**	**Basal-like**
*PIK3CA*	catalytic subunit of PI3 kinase; key signal transduction enzyme involved in cellular growth, survival and insulin signaling	25-36	40-45	29	39	9
*TP53*	tumor suppressor; key regulator of cell cycle, DNA repair, apoptosis	27-37	12	29	72	80
*GATA3*	transcription factor which regulates luminal epithelial cell differentiation in the mammary gland	4-11	14	15	2	2
*MAP3K1*	kinase that activates ERK and JNK kinase pathways	3-8	13	5	4	0
*MLL3*	histone-lysine N-methyltransferase involved in transcriptional co activation	7	8	6	7	5
*CDH1*	cell-cell adhesion glycoprotein; loss-of-function mutations in E-cadherin are a feature of lobular breast cancer	7	9-10	5	5	0

Summary of data from recent breast cancer sequencing studies cited in this mini-review [14-17,20,21].

It is important to note that high-quality next-generation sequencing (NGS) studies are characterized by stringent quality control measures, and study designs that include enough patient samples for accurate assessment of low-prevalence findings. NGS technologies continue to evolve rapidly, driven by the requirement to reduce assay times and cost, while providing sufficient depth and coverage [22].

## The need for repeat tumor biopsies

Clinical studies of tumor heterogeneity at the molecular level, and clonal evolution over time, have been hampered in the past by difficulties in accessing repeated tumor samples from different anatomical areas, and at different time-points. Future studies may be facilitated by two recent developments. First, for patients with metastatic breast cancer, it is increasingly recognized that ER and HER2 status can change over time, and that in selected cases, repeat tumor biopsy is indicated on clinical grounds to determine whether ER or HER2-targeted therapy should be considered [23]. Secondly, it may prove clinically useful to take serial blood samples to sequence circulating tumor DNA (ctDNA), as a less-invasive “liquid biopsy” [24]. New ctDNA assays may have advantages over circulating tumor cells, including greater sensitivity in monitoring tumor response to therapy [25], and a strategy by which to elucidate mechanisms of drug resistance in the clinic [26].

## Conclusion: the best therapeutic strategy. All-out war? Magic bullets? Or uneasy stalemate?

In contrast to metastatic breast cancer, Hodgkin’s lymphoma, testicular cancer and acute myeloid leukemia can be cured using aggressive chemotherapy. However, this is not true for the common metastatic solid tumors. Indeed, high-dose chemotherapy strategies were pursued unsuccessfully by many researchers in an attempt to completely eradicate all cancer cells in a patient’s body [27]. These strategies failed, some authors argue, because of the tremendous genetic heterogeneity of cancers, their spatial dispersion and adaptation to myriad local microenvironments within the individual patient [28]. The suggestion is that for oncologists to achieve the best possible outcomes for their patients, there should be a fundamental change to the canonical treatment approach. Eradication of a dominant, chemosensitive clone may serve only to increase the selective pressure within the tumor, leading to expansion of chemoresistant clones. Rather than killing the maximum number of cells possible, these authors suggest we should be trying to kill the fewest necessary to prevent tumor progression [28]. Others point to drug resistance mechanisms which might be overcome through identification of novel drug targets, the development of new targeted therapies and the rational use of drug combinations [29,30]. One thing, however, is clear; if we are to achieve the promise of personalized medicine, clinical trials will need to follow (with adequate patient numbers) the genetic diversity within tumors which exists in space and time, in relation to the outcomes achieved following different systemic therapies.

## Abbreviations

CNAs: Copy number alterations; CNVs: Copy number variants; ER: Estrogen receptor; ERBB2: Erythroblastic leukemia viral oncogene homolog 2; HER2: Human epidermal growth factor 2; NGS: Next-generation sequencing; SNPs: Single nucleotide polymorphisms.

## Competing interests

The authors declared that they have no competing interests.

## Authors’ contributions

RB and CC jointly wrote the manuscript. Both authors read and approved the final manuscript.

## Authors’ information

RB is Academic Consultant in Experimental Cancer Therapeutics at the University of Cambridge, and Honorary Consultant Medical Oncologist at Cambridge University Hospitals NHS Foundation Trust. His principal research interest is in the development of novel cancer therapeutics and diagnostics for patients with breast cancer.

CC is Professor of Cancer Medicine at the University of Cambridge, Senior Group Leader at the Cancer Research UK Cambridge Institute, and Honorary Consultant Medical Oncologist at Cambridge University Hospitals NHS Foundation Trust. He is also Lead for the Cambridge Experimental Cancer Medicine Centre and Director of the Cambridge Breast Cancer Research Unit.

## Pre-publication history

The pre-publication history for this paper can be accessed here:

http://www.biomedcentral.com/1741-7015/11/151/prepub
